# Participation-performance tension and gender affect recreational sports clubs’ engagement with children and young people with diverse backgrounds and abilities

**DOI:** 10.1371/journal.pone.0214537

**Published:** 2019-04-17

**Authors:** Ramón Spaaij, Dean Lusher, Ruth Jeanes, Karen Farquharson, Sean Gorman, Jonathan Magee

**Affiliations:** 1 Institute for Health and Sport, Victoria University, Melbourne, Victoria, Australia; 2 Department of Sociology, University of Amsterdam, Amsterdam, The Netherlands; 3 Centre for Transformative Innovation, Faculty of Business and Law, Swinburne University of Technology, Melbourne, Victoria, Australia; 4 Faculty of Education, Monash University, Frankston, Victoria, Australia; 5 Department of Education and Social Sciences, Swinburne University of Technology, Melbourne, Victoria, Australia; 6 School of Media, Culture & Creative Arts, Curtin University, Western Australia, Australia; Middlesex University, UNITED KINGDOM

## Abstract

Sport participation has been shown to be associated with health and social benefits. However, there are persisting inequities and barriers to sport participation that can prevent children and young people with diverse backgrounds and abilities from accessing these benefits. This mixed methods study investigated how diversity is understood, experienced and managed in junior sport. The study combined in-depth interviews (n = 101), surveys (n = 450) and observations over a three-year period. The results revealed that a focus on performance and competitiveness negatively affected junior sports clubs’ commitment to diversity and inclusive participation. Gender and a range of attitudes about diversity were also strongly related. On average, we found that those who identified as men were more likely to support a pro-performance stance, be homophobic, endorse stricter gender roles, and endorse violence as a natural masculine trait. In addition, those who identified as men were less likely to hold pro-disability attitudes. These findings suggest that the participation-performance tension and gender affect to what extent, and how, sports clubs engage children and young people with diverse backgrounds and abilities.

## Introduction

Participation in sport can have health and social benefits for both children and adults [1–3]. Motivations for children and young people to participate in sport centre on associated social and health outcomes [1, 4]. On a broader level, sport participation can provide an educational context and foster social interactions that teach children social life skills, sportspersonship, teamwork, and self-efficacy [3, 5–6]. Research suggests that, in certain conditions, sport has the capacity to contribute to social inclusion and, as such, it has become an important site to engage socially vulnerable young people and assist in their psychosocial development [7–8]. However, there are persistent inequities in sport participation [9–11], as well as multilevel barriers that constrain the participation of diverse population groups [12–13]. Historically, organized sport has been a setting where diverse young people have struggled to gain access and develop a sense of belonging, whether it be based on gender [14–17], race/ethnicity [18–20], socioeconomic status [11, 21] or disability [22–25]. These social factors continue to influence disparities in sport participation today.

Providing inclusive sports activities that are safe and welcoming to diverse population groups is critical if access to the health and social benefits of recreational sport is to be broadened and democratized [13, 26]. This study empirically investigated the policy objective of promoting inclusive sports environments for people of all backgrounds and abilities. The aim of this study was to identify how diversity is understood, experienced and managed in junior-age sport. For many children and young people, sport is an important site for socialization where they interact with people with diverse backgrounds and abilities, learn about societal norms and develop a sense of community [27–28].

Demographic diversity occurs when people of varied backgrounds in terms of gender, race/ethnicity, ability or other social factors are present and interact. Diversity is socially constructed: it is a result of the definitions that people in a network of social relations make. In this study, we understood and analysed diversity in relation to their sports club, as a social network or context in which definitions of diversity arose and were used. The study focused on how three forms of diversity that have historically faced discrimination and disadvantage (i.e., gender, cultural diversity, and disability) are understood, experienced and managed by junior sports participants—players, parents, volunteers, committee members and others—within the context of their club and sport. Rather than focusing solely on one type of diversity, the study thus covered a broader spectrum of (intersecting) social relations that are relevant to understanding access to sport participation and associated health and social outcomes [29–30].

## Materials and methods

This study used a mixed methods design that involved four integrated phases conducted over a three-year period [31]:

- Phase 1: 101 in-depth interviews with junior sports participants (committee members, coaches, volunteers, parents and players)
- Phase 2: 450 surveys (pooled analysis)
- Phase 3: Social network analysis of individual clubs
- Phase 4: 200 hours of observations at a sub-sample of junior sports clubs
- Phase 5: Policy analysis of government, peak-body and club policy documents

In this article, we report exclusively on study phases 1, 2 and 4.

**Fig 1:**
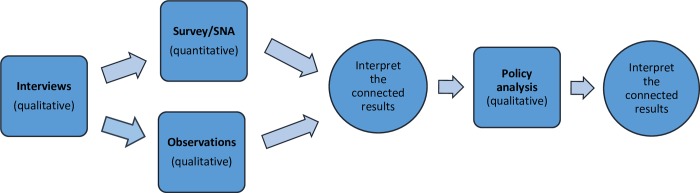
Mixed methods research design [33].

The findings from different research phases and methods were compared systematically [32]. The qualitative methods in phases 1 and 4 provided rich contextual understanding coupled with the broad relationships and patterns among variables uncovered through the survey in phase 2. The survey provided an account of attitudes toward diversity within the clubs, whereas phase 4 (observations) offered a sense of process by examining socialization in ways of experiencing and managing diversity as it unfolds on the ground. Fig 1 summarizes the mixed methods research design, which contained both sequential and parallel data collection and analysis strands.

### Club sampling and recruitment

The research used purposive sampling to select and recruit Australian junior sports clubs for inclusion in the study. Junior sport clubs were defined as not-for-profit, voluntary based organizations that provided sports leagues and competitions to children and young people. Sports clubs are the basis of grassroots participation in organized sport in Australia. Clubs are guided by national and state sport policies as well as state and local government regulations, but have a significant degree of autonomy in their operations. Sports clubs are, in effect, responsible for the practical translation of policy ambitions. State sporting associations and organisations such as VicHealth, a quasi-governmental, statewide health promotion organisation, provide clubs with support and resources to promote inclusive sports provision at the grassroots level.

The clubs (n = 9) were selected based on the following sampling criteria:

- Each club had to organize competitions for children and young people in one or more of five mass participation sports: Australian football, soccer, netball, cricket and basketball;
- Each club had to have at least of 80 registered members (to enable social network analysis);
- Each club had to be recognized by its sports governing body as being actively engaged in diversity and inclusion initiatives, hence constituting “good practices”.

The research team developed a database of clubs that met the sampling criteria, in consultation with the partner organizations (Victorian Health Promotion Foundation, Australian Football League and Centre for Multicultural Youth) and relevant sports governing bodies. The identified clubs were formally invited to participate in the research through an information package that outlined the research aims, methodology and the nature of participation. Club committees formally approved their club’s participation in the project in writing; for de-identification purposes, all clubs were assigned a pseudonym. The sample was diverse with regard to the clubs’ geographical location, types of sport, areas of diversity (gender, cultural diversity, disability) and socio-economic status. The clubs covered five mass participation sports: Australian Rules football, soccer, netball, basketball, and cricket. Seven clubs were located in urban areas; two clubs were located in regional and rural Victoria.

The geographical areas in which the clubs were located ranged from very low to very high in terms of socio-economic status on the Socio-Economic Index for Areas (SEIFA) [34].

### Data collection

#### Interviews

Phase 1 of the study involved a total of 101 face-to-face interviews across the nine clubs. Interviews were conducted at club venues, cafés, workplaces or participants’ homes. Interviews with children and young people ranged from 10 to 20 minutes, while adult interviews ranged from 30 to 90 minutes. Ten per cent of participants were aged 10–14, 20% were aged 15–19, 10% were aged 20–24, 20% were aged 25–34, 20% were aged 35–44, and 20% were aged 45–54. Approximately 60% of interviewees identified as men and 40% as women. The interviews began with questions about respondents’ personal and sporting history, followed by questions that investigated their attitudes to, and perceptions and experiences of, diversity at their club (S1 File). Further questions explored any behaviors or actions respondents had taken to promote diversity at the club or within their own team as well as their perceptions and experiences of resistance to diversity at the club.

#### Survey

This aspect of the research was conducted as a cross-sectional questionnaire (S2 File) that examined individual-level predictors (e.g., gender, age) of various attitudes (e.g., pro-performance, gender equality) that participants within the clubs held, using linear regression models. The selection of the four clubs for this research phase was determined by initial findings from the interviews. Questionnaires were completed at club venues either after training or on game day.

#### Observations

This phase of the research aimed to complement the interviews and survey by providing further insights into how junior sport participants are socialized into ways of managing diversity as it unfolds on the ground. Research relationships with players, parents, coaches and other key club personnel developed within the club setting. Two clubs were invited and agreed to take part in the observation phase of the study. The researchers attended practice sessions, matches, social functions, and club meetings during one full season (six months). A semi-structured observation protocol was developed and implemented (S3 File).

#### Positionality

The researchers’ positioning in relation to the social and political context of the study (i.e., the clubs and their members) is noteworthy. The researchers had had no prior engagement with the clubs, with the exception of some previous research interviews with club committee members at two of the clubs. There was no personal relationship between the researchers and the club communities at the time of the study. The research team was diverse in terms of gender, age, cultural background and academic level. Politically, they were committed to the promotion of safe and inclusive sports environments for children and adults. Throughout the study, the researchers maintained an identity as independent, university-based researchers. In the latter stages of the study, they also actively worked with club representatives to assist in translating research findings and recommendations into practice.

### Qualitative data analysis

The research team employed multiple techniques to enhance the trustworthiness and credibility of the qualitative data. Pilot interviews and observations were initially conducted to develop and test the appropriateness of the interview questions and observation protocol. Based on this pilot, the interview guide was refined and a number of questions were reformulated and added. In addition, ongoing discussion and reflection within the research team allowed for researcher triangulation. Investigators who are experts in specific diversity issues (e.g. race/ethnicity, gender, Indigeneity) provided advice and input into the development of the interview guide and observation protocol. Finally, data triangulation was performed by systematically comparing the qualitative data to the quantitative results.

Interviews were audio recorded and transcribed verbatim, while observations were recorded using field notes. The interview transcripts and field notes were entered into Nvivo 11 data analysis software and coded using thematic analysis techniques. The research team independently read a proportion (10%) of the interview transcripts and field notes. Each investigator coded passages of text firstly using an open (or initial meaning code) and secondly an axial (or categorization of open codes) coding scheme. After similar statements related to a theme were open coded, all the statements under this code were then coded a second time to further categorize the statement. Dialogue among the research team resulted in intersubjective agreement on the interpretation of the identified passages and codes. Two research team members then coded the transcripts line by line. A third investigator reviewed the coding, resulting in a final, agreed-upon set of codes and sub-codes. The resulting coding framework for qualitative data analysis can be found in S6 File. In this specific paper, we focus on a small number of codes that emerged as highly salient during the qualitative data analysis process.

### Statistical analyses of the survey data

The survey data (pooled across all clubs) was analyzed using linear regression models in SPSS in order to identify overall patterns in responses and how these related to demographic details such as gender, ethno-cultural background and disability. Overall, 450 participants (63.1% male; 62.4% under 18 years of age) completed the survey (Table 1). The average age was 21.0 years and the average length of club membership was 4.4 years. The average socio-economic score was 986.89. This socio-economic score is a measure used by the Australian Bureau of Statistics (SEIFA). It is based upon home postcode, with an average of 1000 (and a standard deviation of 100) and higher scores representing higher socio-economic status.

**Table 1:** Survey sample.

	Overall sample
# Survey respondents	450
Adults	169 (37.6%)
Children	281 (62.4%)
Male	284 (63.1%)
Female	166 (36.9%)
Roles	
• Committee	29 (6.4%)
• Parents	16 (3.6%)
• Coaches	29 (6.4%)
• Players	400 (88.9%)
• Other	14 (3.1%)
Sports played	
• Football	197 (44.0%)
• Netball	45 (10.0%)
• Soccer	204 (45.2%)
• > 1 sport	4 (0.8%)
# Disabilities	12 (2.7%)
# Dominant culture	314 (69.3%)
Births overseas	
• Participant	50 (11.5%)
• Mother	139 (30.9%)
• Father	140 (31.1%)
Average age (years)	21.02
Age range (years)	(11–67)
Average socio-economic score	986.89
Socio-economic score range	(831.63–1152.49)

### Ethics approval and consent to participate

The Victoria University Human Research Ethics Committee approved this specific study, the study protocol, and the consent procedures, which adhered to the latest version of the National Statement on Ethical Conduct in Research Involving Humans. Written consent forms were obtained from all participants. Competence to give unspecified consent was determined by the researchers during the introductory conversation (pre-interview) with potential participants (aged 13 and above). During this conversation, all relevant information regarding the project (aims, methods, participants’ rights, etc.) was communicated and, if deemed competent, the participant was asked to sign a consent form. For participants aged 13–17, one parent or guardian provided written unspecified consent, in combination with the written unspecified consent of the underage participant.

## Results

The results revealed a range of experiences of diversity at the nine sports clubs. Multiple factors influenced the way players, parents, board members, volunteers and coaches understood, experienced and managed diversity. The qualitative data showed that understandings of diversity were broad and differed significantly between individuals and clubs [35]. Particular understandings were shaped by club culture and local context; for example, clubs located in neighborhoods with high levels of cultural diversity tended to have greater awareness of and engagement with cultural diversity. Clubs tended to focus on aspects of diversity that were already present within their environments, while often neglecting or marginalising other aspects of diversity. For example, clubs with established girls’ or women’s teams reported that they were effectively supporting diversity despite their lack of consideration of other forms of diversity, such as disability or cultural diversity. The findings also revealed that clubs primarily considered and acted on individual axes of diversity in isolation; they were not inclined to view different forms of diversity as being inter-related. In doing so, their actions were informed by a narrow conception of diversity, with more intersectional understandings of diversity being virtually non-existent within the nine sport clubs.

The results identified two factors that significantly affected to what extent and how junior sports clubs’ engaged with children and young people with diverse backgrounds and abilities: the participation-performance tension, and gender.

### Participation-performance tension

The findings revealed a tension between the promotion of diversity and inclusive participation on the one hand, and the focus on sport performance (i.e., winning and competitiveness) on the other hand. Club members’ attitudes towards participation/ performance significantly influenced their approach to diversity. The data showed that the clubs varied in the degree to which they focused upon participation or performative aspects of sport. However, performance pressures existed at all clubs, albeit in different ways. In some clubs, diversity was afforded less priority than performance. This was evident in, for example, the way these clubs prioritized resource allocation. In the interviews, for example, diversity was often viewed as peripheral to, or diverting resources from, a club’s core business, which revolved around organizing teams and improving playing skills. These clubs thus had a particular understanding of what constituted their core business and what constituted organisational success. For example, a performance-oriented female soccer coach reflected:

“That’s one thing I see the club changing in, it used to be definitely everybody must get an equal time, equal share, blah, blah, blah. But, I do see that the club is starting to turn on, well that’s great, but the main goal is to win, and the main goal is to win premierships. But that is the way the world has gone, and that’s the way society … I wanted to help the club win some premierships; because they haven’t won premierships in a long time.”

Revealing a similar dynamics, a more participation-oriented female netball coach reflected:

“Why do we all have to push our kids you’ve got to win to be great? Someone’s got to come second. And I’m not probably pushy enough […] ‘cause I always think go out there and do your very best and I want you to have a great time. Whereas other coaches, you go to win and winning and we’re getting the championship. So I’m probably not strong enough like that. I’m better probably with littler kids. […] It’s a game of netball for God’s sakes, let’s put into perspective; they’re not going to war. Like some people are very serious about their football and netball around here.”

In the survey and interviews, most clubs positioned themselves towards the participation end of the spectrum, meaning that they identified the core aim of the club as being to provide opportunities to participate in sport. However, the qualitative data revealed that this philosophy was compromised where teams had less ability and faced repeated defeat by opposition teams. For example, two clubs reported that opposing teams’ focus on winning as opposed to giving all children equal playing time led to unsatisfactory experiences for children who lost week in week out. The male president of a soccer club described this tension as follows:

“Overall the club philosophy is about giving people as much time on the pitch as possible […] But some coaches apply that differently and it’s not really policed in that sense, so if some girls are regularly being benched it’s hard to manage that unless parents are telling us and ultimately the coach decides. There will be some pressure put on that coach about fairness, but ultimately they may have a different thing about trying to get as… make their team as competitive as possible. It’s no point playing every week where you’re getting thrashed seven nil if you’ve got half your team, your best players on the bench because you’re doing that spreading that fairly.” [36]

In addition, clubs that actively promoted diversity were generally regarded by outsiders (e.g., coaches and parents from other clubs) as not serious and as having little or no interest in developing talented players. These clubs were therefore perceived as being appropriate for children and young people who were not serious about or “no good” at sport. This labeling process presented challenges for clubs that aimed to provide opportunities for all participants, regardless of ability, and sought to instil a participation rather than performance-based culture. For example, one club had an equal game time policy for all junior players, but when they adhered to this policy and rotated high ability players, coaches received heavy criticism from parents, who argued that the team was not competitive enough.

### Gender

The survey found that gender (i.e., self-identifying as female) significantly predicted pro-participation attitudes (i.e., giving everyone a chance to participate rather than playing to win) [35]. Gender (this time, identifying as man) also predicted adherence to strict gender roles (see Table 2). We further found that those who identified as men, but also younger participants, were more likely to agree with statements regarding masculine violence (e.g., “it’s natural for men to get into fights”). This finding may suggest greater adherence or acceptance by young people more generally for male violent behavior. Regarding homophobia, significant predictors were, again, identifying as men and being younger. Finally, we found that those who identified as women were significantly more likely to hold pro-disability attitudes captured in statements such as “I would be happy to have players with a disability on my team, even if it would limit my team’s chance of success” (see Table 2).

**Table 2:** Linear regression models for participants from four sports clubs (n = 450) predicting pro-participation, strict adherence to gender roles, gender equal treatment within club, endorsement of masculine violence, homophobia and pro-disability attitudes.

	Unstandardized Coefficients		Standardized Coefficients		
	B	Std. Error	Beta	t	Sig.
**Dependent Variable: Pro-Participation**					
(Constant)	4.799	1.414		3.394	< .001
Socio-economic status	-.000	.001	-.005	-.094	.925
Age	.002	.006	.015	.300	.765
Gender (male = 1)	-.578	.149	-.193	-3.866	< .001
Dominant cultural background	-.088	.151	-.028	-.581	.561
**Dependent Variable: Strict adherence to gender roles**					
(Constant)	2.665	1.231		2.164	< .05
Socio-economic status	.000	.001	-.015	-.310	.757
Age	-.016	.005	-.153	-3.188	< .01
Gender (male = 1)	.711	.129	.272	5.514	< .001
Dominant cultural background	.021	.130	.008	.162	.871
**Dependent Variable: Girls treated equally to boys at this club**					
(Constant)	2.844	1.586		1.793	.074
Socio-economic status	.003	.002	.091	1.861	.063
Age	-.011	.006	-.087	-1.784	.075
Gender (male = 1)	.699	.166	.211	4.215	< .001
Dominant cultural background	-.371	.168	-.105	-2.207	< .05
**Dependent Variable: Endorsement of masculine violence as natural**					
(Constant)	4.506	1.561		2.887	< .01
Socio-economic status	-.001	.002	-.028	-.594	.553
Age	-.037	.006	-.277	-5.589	< .001
Gender (male = 1)	.737	.163	.219	4.513	< .001
Dominant cultural background	-.172	.165	-.048	-1.043	.298
**Dependent Variable: Homophobia**					
(Constant)	3.744	1.191		3.143	< .05
Socio-economic status	-.002	.001	-.078	-1.711	.088
Age	-.015	.005	-.144	-3.179	< .01
Gender (male = 1)	1.069	.125	.400	8.575	< .001
Dominant cultural background	-.060	.126	-.021	-.473	.636
**Dependent Variable: Pro-disability**					
(Constant)	8.339	1.701		4.902	< .001
Socio-economic status	-.003	.002	-.085	-1.725	.085
Age	-.005	.007	-.034	-.689	.491
Gender (male = 1)	-.662	.178	-.188	-3.720	< .001
Dominant cultural background	.148	.180	.039	.818	.414

The qualitative data provided further insight into the role of gender [35]. The sports clubs reported that they attempted to create a welcoming and inclusive environment for girls and women, yet some clubs revealed persistent gender divisions and a male-dominated atmosphere. For example, referring to gender differences at her club, a female soccer coach stated: “The same as at every club, they have to really fight to get the same recognition as the men. I’ve always seen that, and always felt that. They could be as committed as anything, and prove their worth, but because they don’t draw the crowd that the men draw they aren’t treated as they are as important to a club as the men are.” Several respondents who identified as women reported that they did not attend club social events because they felt uncomfortable in those spaces. For example, a netball coach reported that “it is not that welcoming or friendly, I never really feel like I should be there.” The club facilities were frequently decorated with memorabilia and cultural artefacts (e.g., trophies, photographs, posters) that highlighted the men’s teams’ successes rather than the accomplishments of girls’ and women’s teams.

## Discussion

In line with previous studies of diversity in everyday discourse [37], this research has found that diversity was interpreted in many different, yet often abstract and universal, ways within and across the junior sports clubs. Individuals were frequently confused about the language of diversity, how diversity actually related to them within the context of their club, and the attitudes or behaviors associated with it. The particular context and culture of each club shaped the parameters for how diversity was understood and experienced, which in turn dictated practical responses to diversity within each club. This finding is consistent with recent research which suggests that the contexts of sports clubs influence how they respond to policy objectives [38].

The clubs in this study demonstrated varying levels of commitment to diversity. Overall, there was not a consistent approach to the promotion of diversity and inclusion of people of all backgrounds and abilities, nor was there consensus across the clubs that inclusive participation was an important objective. Most clubs recognized the benefits of diversity in terms of increased club membership, additional volunteers, sustainability, and social and health benefits to participants. This finding aligns with management literature that shows how organisations can enact both a business and a social justice case for diversity [39]. Yet, in contrast to previous studies which found that belief in the benefits of diversity results in higher levels of diversity management practice [40], this research revealed that junior sports club leaders and members frequently felt overwhelmed by, and under-resourced to deliver on, policy calls to actively promote diversity and social inclusion. Most clubs felt that promoting diversity was beyond their means. Clubs and their volunteers were not necessarily resisting diversity but were implementing diversity in (restricted) ways that they believed the club could cope with. This finding confirms the conclusion of previous research that volunteers face considerable pressure within sports clubs, particularly when delivering agendas that are not see as a core focus [41–42].

The findings complement previous studies that highlight the importance of individual and organizational commitment to diversity for organizational change and creative work environments in sport [43–46]. Two findings stand out. First, our results showed that gender and a range of attitudes about diversity are strongly related. On average, we found that those who identified as men were more likely to support a pro-performance stance, be homophobic, endorse stricter gender roles (while believing there is gender equality) and endorse violence as a natural masculine trait. In addition, those who identified as men were less likely to hold pro-disability attitudes. This does not mean that all individuals who identified as men endorsed such views at levels greater than all who identified as women, but that in general men’s responses were higher on average than women’s responses. These results are consistent with research on gender differences using economic games and moral dilemmas. This body of research indicates that women are more altruistic than men in Dictator Game experiments [47–48], somewhat more cooperative than men in social dilemmas [49], and more harm averse than men in personal moral dilemmas [50].

Contextual factors may have driven the attitudes of those who identified as men in some clubs in certain directions. Indeed, differences between clubs highlight the impact such contextual factors may have had [35]. For example, clubs varied in terms of the socioeconomic, age and cultural diversity of its membership and leadership, which interacted with gender to shape specific attitudes to diversity in complex ways. Beyond demographics, organizational culture played a role in shaping attitudes and behaviors concerning diversity, such as to what extent leaders afforded priority to inclusive participation within club structures, practices and resources. Nonetheless, the findings suggest that gender can intersect with a range of diversity attitudes and can be a facilitator or constraint to diverse and inclusive participation within sports clubs. The particular context of a sports club is likely to moderate these effects. Previous research has indicated how gender shapes girls’ and women’s participation in sport [14–17], but few studies have explored how gender mediates attitudes toward other forms of diversity. Our findings suggest that having more people who identify as women involved in the club, particularly in leadership and management positions, can affect club attitudes and practices toward diverse and inclusive participation. A second major finding is that attitudes of junior sports clubs and their members in regards to participation or performance significantly influenced how they understood and managed diversity. This finding reflects the documented tension between power/performance and pleasure/participation ideologies in global sport [51–52], suggesting that these ideologies also trickle down to the junior level.

The findings reported in this article were drawn from a purposive sample: a set of junior sports clubs that were recognized by their sports governing bodies as being relatively active and successful in promoting diverse participation. It could be hypothesized that other sports clubs, including those that do not engage with diversity or that resist efforts at diversification, experience comparable or more accentuated issues regarding the engagement and inclusion of people with diverse backgrounds and abilities. The current study expands upon existing knowledge on barriers to sport participation experienced by children and young people with diverse backgrounds and abilities. Our study warrants further research investigating the effects of pro-performance attitudes and practices, and gender relations on inclusive participation in recreational sports clubs, taking into consideration the present limitations and suggestions made.

## Supporting information

S1 File Interview protocol. Interview questions used in phase 1 of this study.
(PDF)

S2 File Survey. Questionnaire used in phase 2 of this study.
(PDF)

S3 File Observation protocol. Observation guide used in phase 4 of this study.
(PDF)

S4 File Data matrix. Data matrix in SPSS format used for the statistical analyses in this study.
(SAV)

S5 File Regressions. Regression analyses in SPSS format.
(SPS)

S6 File Coding framework. Codes used in qualitative data analysis.
(XLSX)
