# Supplementary material for: Participation-performance tension and gender affect recreational sports clubs’ engagement with children and young people with diverse backgrounds and abilities
Source: PLoS One. 2019 Apr 17;14(4):e0214537. doi: 10.1371/journal.pone.0214537 (PMC6469765; doi:10.1371/journal.pone.0214537)
Supplement: S1 File — Interview questions used in phase 1 of this study. (PDF) [file pone.0214537.s001.pdf]

Diversity in Community Sports Clubs: Interview questions

Part 1: General introductory questions

Where are you from/Where did you grow up?

How would you describe your family background (class, socially, culturally, religious?)

Describe what the suburb/town that you grew up in was like?

How would you describe who you are? Ethnically, religiously, economically, socially?

Part 2: General sport questions

What was your first experience of playing sport?

When did you first start becoming interested in sport?

Why was this the case?

How important was sport in your childhood?

Part 3: The individual

How would you describe what diversity is at the club?

Did you have any interactions with people from different backgrounds growing up? What kinds of backgrounds?

What were those relationships like?

Did you have any interactions with people with disabilities growing up? What were those relationships like?

What kinds of experiences have you had with people from different backgrounds at the club?

Do you feel that everyone is treated the same?

Are men and women treated equally?

Are people from different cultural backgrounds treated differently?

Has the club had anyone with any kind if disability? What was it? How were they treated?

What benefits have you personally experienced from people of different backgrounds being at the club? Explain.

What benefits has your club/team experienced from people from different backgrounds?

Have you or your club/team experienced any disadvantages from people with different backgrounds

What things have you or the club done to make people from different backgrounds feel comfortable at the club (or in your team)?

Have people from diverse backgrounds shaped your coaching or management style? If so, how? **(Coaches, club management only)**

Part 4: The club
(Adults only: volunteers, parents, coaches, committee members)

Are you aware of any policies or codes that the club has in place to promote a safe and inclusive sporting environment?

How does the club engage members about these policies? (meetings, flyers, emails, special events, etc.)

What things has the club done to increase awareness about diversity?

Do you think the club's efforts around those initiatives have changed the club at all? If so, how?

What sort of environment do you feel the club provides for people from diverse backgrounds?

Do you think the club should play a role in teaching players/coaches about tolerance and prejudice or should it happen in other areas? Where?

What facilities or policies does the club have in place to support disabled persons to participate? Do you think these are sufficient?

Some sports clubs focus on giving all players a go in games, while others focus on playing to win. If we think of the two as ends of a spectrum, where on the spectrum (between everyone participating and focusing on winning) where would you say your club sits?

Part 5: The sport organisation
(Committee members, coaches, volunteers)

What do you think about the way [your sport organisation] has tried to deal with issues around race, ethnicity or religion?

Have the vilification rules that are in place changed the club/team at all?

How prominent was vilification at the club before the rules were brought in? What are your recollections of it?

Have there been any issues that you are aware of at your club that the vilification laws have helped deal with?

Do you think that the vilification rules have meant that more multicultural players are a part of the game? Why?

What do you think about the way [your sport organisation] has tried to deal with issues around women, sexuality and homophobia?

Part 6: The government
(Committee members, coaches, volunteers)

What do you think about the way the government has tried to deal with issues around diversity in sport, for example the new code of conduct for community sport?

Are you aware of government initiatives such as the code of conduct and junior sport framework? If yes: What things has the club done to engage club members about the government policies that are in place?

Have the government's efforts changed the club at all? If so, how?
