# Supplementary material for: Participation-performance tension and gender affect recreational sports clubs’ engagement with children and young people with diverse backgrounds and abilities
Source: PLoS One. 2019 Apr 17;14(4):e0214537. doi: 10.1371/journal.pone.0214537 (PMC6469765; doi:10.1371/journal.pone.0214537)
Supplement: S2 File — Questionnaire used in phase 2 of this study. (PDF) [file pone.0214537.s002.pdf]

Diversity in Community Sports Clubs Survey (A)

INSTRUCTIONS

You will be asked questions about your opinions, but also about your social connections to other people at your club. When asked about other people (e.g., who are your closest friends?), write the number next to their name _from the list provided.

For Example: Who is the tallest player(s) at the club? ________13________________15________

If someone is not listed, just write their name:

Club ID ☐☐☐ This is your number on the list handed out with this survey.

If you are not on the Club List, **please write your name here**: ______________________________________

Your answers to all questions are totally confidential and will not be disclosed in any way that will identify you or your club. Nobody from your club will see your answers.

Section A: Your details

In this section we would like to get some general information about you.

1. Age ________
2. Male ☐ Female ☐
3. Cultural background: ____________________________
   (e.g., Anglo-Australian, Indigenous, Afghani, Italian-Australian, etc….)
4. Religion: ____________________________
5. Which suburb do you live in? ____________________ Postcode: ____________
6. Were you born in Australia or overseas? ☐ Australia ☐ Overseas
7. Was your mother born in Australia or overseas? ☐ Australia ☐ Overseas
8. Was your father born in Australia or overseas? ☐ Australia ☐ Overseas
9. Which language is mostly spoken at your home? ____________________________
10. What is your role at this club? (Please tick all that apply)

    ☐ Committee member ☐ Player

    ☐ Parent ☐Other ______________

    ☐ Coach
11. Approximately how long have you been involved with this club? _______months _______years
12. Do you have a disability and/or impairment? If yes, please specify ____________________

Section B: People at this club

In this section we are interested in people you interact with at the club. Thinking about your club at the moment, use the list to answer these questions below. Do NOT use any names, just ID numbers (see example below). ***There is no right number of people, you can list as many as you like.***

For Example:<br>*(A). Who is the happiest person at the club? ________ 18 ______ 6 __________*

1. Who are the best players at the club? ____

2. Who are your closest friends at the club? ____

3. Which people at this club do you have different views or opinions to? ______________________

4. Are you related to anyone at the club? If so, who? _____________________________________

5. Who do you most trust at this club (e.g., to drop your kids home from training)?
   ____

6. Who do you provide support to at this club (e.g., give encouragement, a pat-on-the-back, etc..)?
   ____

7. Who sets the culture at this club?____

Section C: You and the club

Circle the number that represents your opinion to the following, with regard to the club

	Strongly Disagree						*Strongly Agree*
1. My opinion doesn't count for much	1	2	3	4	5	6	7
2. Other people listen to what I have to say	1	2	3	4	5	6	7
3. I feel isolated because of my opinions	1	2	3	4	5	6	7
4. I feel comfortable at the club	1	2	3	4	5	6	7
5. The club supports me.	1	2	3	4	5	6	7
6. The club needs to get better at dealing with players as individuals	1	2	3	4	5	6	7
7. The club works well together	1	2	3	4	5	6	7
8. When someone criticizes this club, it makes me really upset	1	2	3	4	5	6	7
9. My family thinks this club looks after me	1	2	3	4	5	6	7
10. This club encourages people to learn about others who are different	1	2	3	4	5	6	7

Section D: Your views

The following statements are about diversity. ***There are no right or wrong answers*** *we are simply interested in how much you* ***agree*** *with each of the following statements. Please use the following scale to indicate how strongly you* ***agree*** *or* ***disagree*** *with each statement.*

1. My club is supportive of people from different cultural and ethnic backgrounds.

Strongly disagree	*Disagree*	*Slightly disagree*	*Neither*	*Slightly agree*	*Agree*	*Strongly agree*
1	2	3	4	5	6	7

2. A person's race determines to a great extent what kind of person they are.

Strongly disagree	*Disagree*	*Slightly disagree*	*Neither*	*Slightly agree*	*Agree*	*Strongly agree*
1	2	3	4	5	6	7

3. Girls/women and boys/men are treated the same at this club.

Strongly disagree	*Disagree*	*Slightly disagree*	*Neither*	*Slightly agree*	*Agree*	*Strongly agree*
1	2	3	4	5	6	7

4. I am uncomfortable around people who are different from me.

Strongly disagree	*Disagree*	*Slightly disagree*	*Neither*	*Slightly agree*	*Agree*	*Strongly agree*
1	2	3	4	5	6	7

5. When playing in or spectating my team I get annoyed with some players who are not as talented as others.

Strongly disagree	*Disagree*	*Slightly disagree*	*Neither*	*Slightly agree*	*Agree*	*Strongly agree*
1	2	3	4	5	6	7

6. I would be happy to have players with a disability on my team, even if it would limit my team's chance of success.

Strongly disagree	*Disagree*	*Slightly disagree*	*Neither*	*Slightly agree*	*Agree*	*Strongly agree*
1	2	3	4	5	6	7

7. Rather than give all players equal game time, we should always field the strongest team possible so we have the best chance of winning each week.

Strongly disagree	*Disagree*	*Slightly disagree*	*Neither*	*Slightly agree*	*Agree*	*Strongly agree*
1	2	3	4	5	6	7

8. It is OK for a man to cry in public

Strongly disagree	*Disagree*	*Slightly disagree*	*Neither*	*Slightly agree*	*Agree*	*Strongly agree*
1	2	3	4	5	6	7

9. I am accepting of gay men.

Strongly disagree	*Disagree*	*Slightly disagree*	*Neither*	*Slightly agree*	*Agree*	*Strongly agree*
1	2	3	4	5	6	7

10. A woman should expect to do most of the childcare.

Strongly disagree	*Disagree*	*Slightly disagree*	*Neither*	*Slightly agree*	*Agree*	*Strongly agree*
1	2	3	4	5	6	7

11. It's natural for men to get into fights

Strongly disagree	*Disagree*	*Slightly disagree*	*Neither*	*Slightly agree*	*Agree*	*Strongly agree*
1	2	3	4	5	6	7

12. It is disappointing to learn that a famous athlete is gay.

Strongly disagree	*Disagree*	*Slightly disagree*	*Neither*	*Slightly agree*	*Agree*	*Strongly agree*
1	2	3	4	5	6	7

13. A man should force the issue if another man takes his parking place

Strongly disagree	*Disagree*	*Slightly disagree*	*Neither*	*Slightly agree*	*Agree*	*Strongly agree*
1	2	3	4	5	6	7

14. Some sports clubs focus on giving all players a go in games, while others focus on playing to win. If we think of the two as ends of a spectrum, where on the spectrum (between everyone participating and focusing on winning) where would you say your club sits?

Everyone gets a go									*Playing to win*
1	2	3	4	5	6	7	8	9	10

Section E: Your Wellbeing

1. In general, would you say your health is…?

Poor	*Fair*	*Good*	*Very good*	*Excellent*
1	2	3	4	5

2. Thinking about your own life and your personal circumstances please rate how satisfied you are with…

		Completely Dissatisfied									*Completely Satisfied*
a.	Your life as a whole	1	2	3	4	5	6	7	8	9	10
b.	Feeling part of your community	1	2	3	4	5	6	7	8	9	10
c.	Feeling part of your sports club	1	2	3	4	5	6	7	8	9	10

3. How safe or unsafe do you feel <u>your children are</u> when they are in the following situations?

How safe do you feel your children are…?

		Very unsafe	*Unsafe*	*Neither safe nor unsafe*	*Safe*	*Very Safe*
a.	On game day at this club?	1	2	3	4	5
b.	On game day at other clubs?	1	2	3	4	5
c.	Training at this club?	1	2	3	4	5
d.	At club social functions?	1	2	3	4	5
e.	Walking in your local area alone during the day?	1	2	3	4	5

Other Comments

Please feel free to make any comments about this survey or share your thoughts on issues raised

____

____

____

____

This completes the survey - please spend a moment to make sure that you haven't missed any questions.

THANK YOU FOR YOUR TIME
