# Supplementary material for: Participation-performance tension and gender affect recreational sports clubs’ engagement with children and young people with diverse backgrounds and abilities
Source: PLoS One. 2019 Apr 17;14(4):e0214537. doi: 10.1371/journal.pone.0214537 (PMC6469765; doi:10.1371/journal.pone.0214537)
Supplement: S3 File — Observation guide used in phase 4 of this study. (PDF) [file pone.0214537.s003.pdf]

Diversity in Community Sports Clubs: Participant Observation Protocol

(Guidance for Research Assistant)

Purpose of participant observations and relationship with other research methods

The participant observations are intended to provide the research team with a description of what actually happens within the club environment in relation to issues of diversity. Within the project we are seeking to understand how diversity is treated and understood. Watching how key actors (junior player, parents, coaches, club committee) conduct their 'day to day' activity in the club environment is a critical part of this. The project aims to produce detailed data on how junior sports participants are socialised into ways of managing diversity as it unfolds on the ground. The participant observation data should provide concrete examples of how the relationships that are mapped via the Social Network Analysis play out in practice in the live club environment. They will likely also provide tangible examples of how various actors in clubs interact in ways that are spoken about in the interviews.

What will be observed?

The research team would like the researcher to observe 'normal activity' within the club environment. This will likely consist of junior team training sessions, competitive matches and tournaments, club committee meetings (where the club is agreeable). It may also include club social functions, junior player recruitment days, coaches meetings or training. Obviously the setting will influence what is being observed. The key overarching aspect we are looking to observe is how do individuals in the club interact with each other and how do elements of diversity mediate these interactions?

Template for Observing Sessions:

What type of session is being observed? (i.e. training, match)	
Who is in attendance? (i.e. number of junior players, coaches, parents, etc.)	
Brief outline of the focus/intention of the session (i.e. if a meeting what is the meeting about/ training what is being covered)	
Are there any observable indications of diversity in the setting observed? (i.e. gender balance/ ethnicity/disability)	
Are there any observable indications of awareness of diversity issues in the setting observed? (i.e. Where (if at all) is the code of conduct displayed? Are there any signs or slogans that emphasise the importance of participation or winning (e.g. 'Whatever it takes'-type slogans)? Any images that could be considered sexist? What facilities are in place for people with physical disabilities?)	
Interactions between participants: please make notes on how individuals interact with each other during this setting. In particular we want to focus on who individuals interact most with and who they don't interact with. For the latter it would be helpful to indicate whether you feel issues of diversity are impacting on this. For example, if during a committee meeting one member is not contributing very much and they are also female we need to know it was the female member not contributing.	
Interactions between junior players (How do the players work together? Do particular players tend to stick with certain others? Who seem to be the leaders amongst the players?)	
Interactions between junior players and coaches (How does the coach interact and who with? Do particular players get more attention than others? What is the coaches' style and approach? Do coaches do anything differently for certain individuals? i.e. if disabled and mainstream together how is this accommodated)	

Interactions between junior players and parents (How do parents behave at training, matches, etc.? Do they get involved in the training at all? Do they interact with others players beyond their own children?)	
Interactions between coaches and parents<br>(Do coaches speak with parents before/during/after sessions? Do they talk to particular parents?)	
Interactions between committee members (who appears to be the main leaders/dominant members of the committee? What is the committee make up? Who tends to influence/make most of the decisions? Do any issues regarding diversity emerge during meetings?)	
Interactions between committee members and coaches (what sort of interactions take place between these groups, what types of issues are raised? What sort of support do coaches get from the committee?)	
Other interactions	
Within these interactions, did you feel you observed any examples of discrimination? This could include overt examples like derogatory comments, players actively excluding another player during activities. Or it may be more subtle, coaches giving less feedback to particular players, the views of certain committee members rarely being sought or being dismissed.	
When observing these interactions did you witness any examples of obvious effort to manage and include diverse groups? This could range from things like club committee members seeking the views of all members, discussing how best to implement inclusive policies through to coaches adapting drills and activities so that low ability/disabled individuals could take part.	
Other observations that you deem relevant to the project	
